# A scoping review of biomechanical testing for proximal humerus fracture implants

**DOI:** 10.1186/s12891-015-0627-x

**Published:** 2015-07-30

**Authors:** David Cruickshank, Kelly A. Lefaivre, Herman Johal, Norma J. MacIntyre, Sheila A. Sprague, Taryn Scott, Pierre Guy, Peter A. Cripton, Michael McKee, Mohit Bhandari, Gerard P. Slobogean

**Affiliations:** Department of Orthopaedics, University of British Columbia, Centre for Hip Health & Mobility, Robert H.N. Ho Research Centre, 771 - 2635 Laurel Street, Vancouver, BC V5Z 1 M9 Canada; School of Rehabilitation Science, McMaster University, Hamilton, Ontario Canada; Division of Orthopaedic Surgery, Department of Surgery, McMaster University, Hamilton, Ontario Canada; Department of Clinical Epidemiology and Biostatistics, McMaster University, Hamilton, Ontario Canada; Department of Mechanical Engineering, University of British Columbia, Vancouver, British Columbia Canada; Division of Orthopaedic Surgery, Department of Surgery, University of Toronto, Toronto, Ontario Canada; Department of Orthopaedics, University of Maryland School of Medicine, Baltimore, Maryland USA

**Keywords:** Proximal humerus fracture, Biomechanics, Proximal humerus fracture implant

## Abstract

**Background:**

Fixation failure is a relatively common sequela of surgical management of proximal humerus fractures (PHF). The purpose of this study is to understand the current state of the literature with regard to the biomechanical testing of proximal humerus fracture implants.

**Methods:**

A scoping review of the proximal humerus fracture literature was performed, and studies testing the mechanical properties of a PHF treatment were included in this review. Descriptive statistics were used to summarize the characteristics and methods of the included studies.

**Results:**

1,051 proximal humerus fracture studies were reviewed; 67 studies met our inclusion criteria. The most common specimen used was cadaver bone (87 %), followed by sawbones (7 %) and animal bones (4 %). A two-part fracture pattern was tested most frequently (68 %), followed by three-part (23 %), and four-part (8 %). Implants tested included locking plates (52 %), intramedullary devices (25 %), and non-locking plates (25 %). Hemi-arthroplasty was tested in 5 studies (7 %), with no studies using reverse total shoulder arthroplasty (RTSA) implants. Torque was the most common mode of force applied (51 %), followed by axial loading (45 %), and cantilever bending (34 %). Substantial testing diversity was observed across all studies.

**Conclusions:**

The biomechanical literature was found to be both diverse and heterogeneous. More complex fracture patterns and RTSA implants have not been adequately tested. These gaps in the current literature will need to be addressed to ensure that future biomechanical research is clinically relevant and capable of improving the outcomes of challenging proximal humerus fracture patterns.

**Electronic supplementary material:**

The online version of this article (doi:10.1186/s12891-015-0627-x) contains supplementary material, which is available to authorized users.

## Background

Proximal humerus fractures (PHF) are a challenging injury in need of more reliable surgical techniques and improved health-related outcomes. Intra-articular screw penetration, loss of reduction, and fracture healing complications frequently occur and have limited the success of surgical management [1]. Furthermore, the outcomes associated with three- and four-part fracture patterns are often both unpredictable and worse than anticipated [1–8]. The complications and long recovery times for PHFs have a significant impact on patient quality of life [4, 5, 9] and the health care system [10].

Biomechanical modeling provides controlled testing data to support new surgical implants and novel treatment strategies. Biomechanical research is an important method of evaluating orthopaedic implants as it removes patient factors and focuses on the performance of the implant under strict testing conditions. There has been an increasing focus on biomechanical modeling to test the properties and limits of various techniques and implants used to treat proximal humerus fractures. Since there are numerous surgical implants and PHF patterns that could be tested, the biomechanical literature is potentially a broad landscape of diverse research that has not been previously summarized.

The purpose of the current study was to: 1) use scoping review techniques [11, 12] to systematically evaluate and map the breadth of proximal humerus fracture biomechanical testing literature; 2) to summarize the model designs and testing procedures most commonly employed; and, 3) to identify biomechanical areas that are not well represented in the existing literature.

## Methods

### Literature search

As part of our larger proximal humerus fracture scoping review (Slobogean et al., [13]), we completed a comprehensive literature search to identify studies on the management of proximal humerus fractures. In consultation with a biomedical librarian, we developed a sensitive search strategy to identify all types of publications involving proximal humerus fractures. Using a combination of keywords and medical subject heading (MeSH) terms related to proximal humerus fractures, we searched the following electronic databases: Medline, Excerpta Medica Database (EMBASE), Cumulative Index of Nursing and Allied Health Literature (CINAHL), Cochrane Database of Systematic Reviews (CDSR), Proquest, Web of Science, Society of Automotive Engineers (SAE) digital library, and Transportation Research Board’s Transport Research International Documentation (TRID) database. All searches were performed in October 2012, and no language or date restrictions were employed.

### Study selection

All identified titles were then compiled into a literature review program (*DistillerSR)*, and an independent review process was performed. All studies were reviewed in duplicate by two orthopaedic surgeons, and studies involving biomechanics were identified. We excluded review articles, computer modeling, finite element analysis studies, and studies that were not published in English.

### Data abstraction

Two authors (DC and TS) independently abstracted data from each included study focusing on the characteristics of the analysis and the methods utilized to better understand the layout of the literature. Any disagreements on the data abstracted were resolved by consensus in consultation with a third author (GPS). Study characteristics abstracted included publication year, geographic location, sample size, and type of specimen. Methods data abstraction examined pretesting analysis, implant selection, and testing conditions.

### Statistical analysis

Descriptive statistics were used to summarize all data. For continuous data, the mean and standard deviation or median and ranges were reported based on the data’s distribution. Counts and proportions were used to describe all other data. No inferential statistical testing was performed.

## Results

### Literature review

**Fig. 1:**
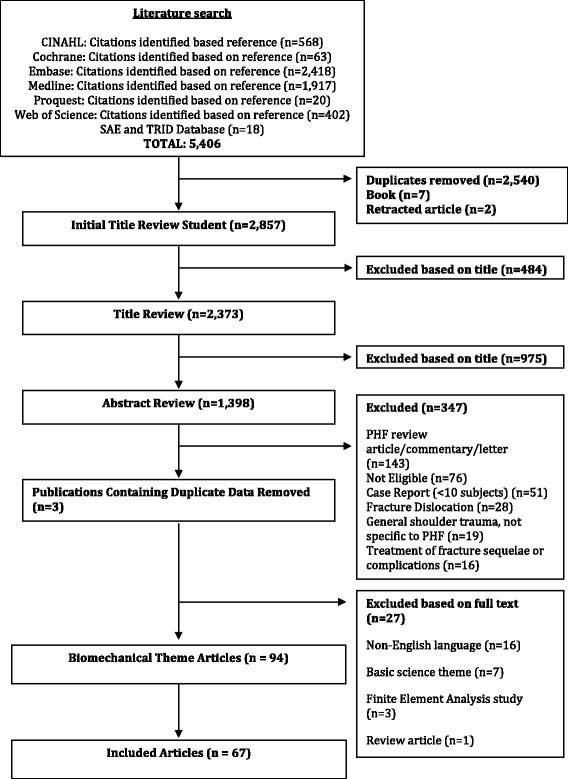
Search and screening flow chart

The initial literature search of the PHF literature, which included clinical and basic science studies, resulted in identification of 5,406 titles. 2,540 were found to be duplicates, seven were book titles, and two were retracted studies; these were all excluded. An additional 1,459 titles were removed because they did not meet the eligibility criteria. After review, 1,051 proximal humerus fracture studies were included in our database. From our eligible PHF database, 94 were identified as basic science or biomechanical papers. For the purpose of this study, we excluded an additional 16 non-English language publications, seven basic science articles, three finite element analysis studies, and one review article (Fig. 1). Therefore, 67 proximal humerus biomechanical published studies were included in the current analysis (Additional file 1).

### Study characteristics

**Fig. 2:**
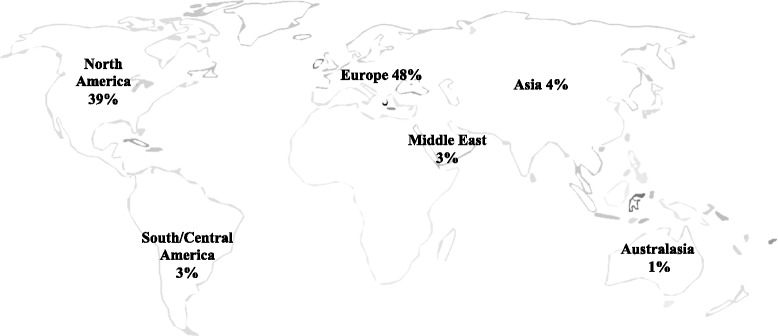
Location of research

**Fig. 3:**
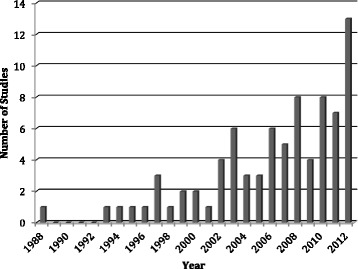
Frequency of studies published per year

The majority of the included publications originated from Europe (48 %) to North America (39 %), comprising 87 % of the total studies. Few biomechanical studies have been published from Asia (4 %), South/Central America (3 %), to the Middle East (3 %) (Fig. 2). The earliest included study identified dates back to 1988 with nothing published again until 1993. Since that time, however, there has been an exponential increase in biomechanical publications, with 13 studies published in 2012 alone (Fig. 3).

### Specimen characteristics

The sample size was reported in every study and the average sample size was found to be 27 ± 28.9 specimens with a range of 5 to 150 specimens (Table 1). The most commonly tested specimen was cadaver bones (87 %) followed by saw bones (7 %), animal bones (4 %), and wood (1 %). Of the cadaver studies, the obtained cadavers were frozen in 45 studies (75 %), embalmed in 12 (20 %), and fresh in 3 (5 %). Of the 58 studies that used cadaver specimens, only 33 (57 %) included information on the age of the cadavers used. In the studies that reported age of the cadaver, the average age was found to be 73.3 ± 8.5 years, with a range of 32 to 101 years of age. Only 45 studies (67 %) undertook some form of pre-testing investigations including plain radiographs (32 studies), bone mineral density testing (31 studies), and CT scans (12 studies) (Table 2).

**Table 1:** Specimen characteristics

Characteristic	Frequency
	N (%)
Type of Specimen (n = 67)	
Cadaver	58 (87)
Saw Bones	5 (7)
Animal	3 (4)
Wood	1 (1)
Cadaver State (n = 58)	
Frozen	44 (76)
Embalmed	11 (19)
Fresh	3 (5)
Fracture Pattern (n = 60)	
2-part	41 (68)
3-part	14 (23)
4-part	5 (8)
Cadaver Age (Mean ± Standard Deviation)	73 ± 8.5
Number of specimens (Mean ± Standard Deviation)	27 ± 28.9

**Table 2:** Testing characteristics

Characteristic	Frequency
	N (%)
	(n = 67)
Pre-Testing Investigations	
Plain radiographs	32 (48)
Bone mineral density testing	31 (46)
CT scans	12 (18)
Testing Constructs	
Torque	34 (51)
Axial load	30 (45)
Cantilever bending	23 (34)
Testing Parameters	
How the force was applied	64 (96)
Loading mode	54 (81)
Magnitude of force	20 (30)
Loading	
Cyclic loading	33 (49)
Load to failure	28 (42)
Compounding cyclic load to failure	6 (9)

Sixty studies tested proximal humerus fracture implants in a specific simulated fracture pattern. The most common fracture simulated was a two-part proximal humerus fracture in 41 studies (68 %), followed by a three-part fracture in 14 studies (23 %), and a four-part fracture in five studies (8 %) (Table 1). Of the two-part fracture simulations, 37 involved the surgical neck, three involved the anatomic neck, and one study described making a two-part fracture model, but the location of the osteotomy was not stated. None of the included studies examined fractures of the greater tuberosity.

The most common method of specimen preparation was to create the fracture using a saw, followed by reduction and fixation with the specified construct. Often, in order to simulate medial comminution, a section of bone would be removed and a gap created. This modification of the specimen ensured that reduction and alignment was maintained solely by the implant in the absence of a medial cortical support. This was performed in 35 studies. When compared to fracture type, medial comminution was simulated in 27 (66 %) of the two-part fracture studies, in seven (50 %) of the three-part fracture studies and in none of the four-part fracture studies. One study specified that a gap osteotomy was performed but did not specify the location or the fracture pattern.

### Implants evaluated

**Fig. 4:**
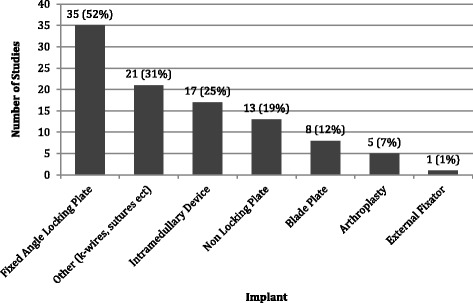
Frequency of implant testing

The most frequently tested implant was a fixed angle locking plate, which was tested in 35 studies (Fig. 4). Intramedullary devices, including intramedullary nails, were tested in 17 studies, followed by non-locking plates in 13 studies, and blade plates in eight studies. Interestingly, arthroplasty implants were only tested in five studies and only included hemi-arthroplasty implants. We did not identify any studies that focused on biomechanical testing of total shoulder implants or reverse total shoulder implants for the treatment of proximal humerus fractures. An overview of the implants evaluated in each study is found in Additional file 2.

### Construct testing

The apparatus and testing procedure of the constructs was found to be highly heterogeneous between studies and many different testing platforms, configurations, and devices were described in the included studies. Despite this heterogeneity, the majority of the studies tested their constructs under similar biomechanical themes, which has allowed us to summarize them. Specifically, the most commonly tested force was torque (34 studies), followed by axial load (30 studies), and cantilever bending, usually in varus or valgus (23 studies) (Table 2).

The testing parameters including the magnitude of the force (20 studies), how the force was applied (64 studies), and the loading mode (54 studies); all testing parameters varied significantly between studies (Table 2). Cyclic loading was utilized in 33 studies, load to failure was used in 28 studies, and compounding cyclic load to failure was used in six studies. In the studies that used cyclic loading to test the construct, the number of cycles varied from 5 to 1,000,000; the most commonly used number of cycles was 1000 (seven studies). Many studies (34 studies) used a combination of testing modes, for example a construct would be put through cyclic loading to a set number of cycles and then undergo a load to failure test.

Supplementary fixation methods were infrequently evaluated in the biomechanics literature. Sutures were used to augment fixation in three studies; two of the studies tested hemi-arthroplasty implants and one study tested locking plates. Tension band wiring, either on its own or as an augment to a construct, was used in four studies. Bone grafting with structural grafts was tested in three studies and two studies examined the use of cement as an augment.

## Discussion

The literature describing the biomechanical testing of proximal humerus fracture implants is broad and heterogeneous. It is evident that biomechanical testing is being performed more frequently to compare proximal humerus fracture treatments; however, significant limitations to the clinical utility of the current testing models exist. These include a lack of models for three- and four-part fractures and a high variability in the testing parameters utilized.

The most common model identified was the simulated two-part fracture. From a practical perspective, this is not surprising since the fracture (osteotomy) occurs in the surgical neck region and does not require the investigator to recreate fractured tuberosity fragments or impaction of the humeral head. Two-part fractures are also appealing to model because fixation is easily achieved in the humeral head and shaft, and mechanical testing can focus on axial, bending, and torsional loads across a single fracture line. Despite the study design advantages of focusing on two-part fractures, it is our opinion that three- and four-part fractures represent the true surgical challenge and should be the focus of most biomechanical testing [7, 8]. Fourteen studies simulated a three-part fracture, and only five studies used a four-part model.

Another key finding of our scoping review was the substantial heterogeneity in testing parameters. We found almost no duplication of testing configurations and minimal standardization, which would allow comparison between studies. Consequently, we classified the studies based on biomechanical testing themes such as direction of force and testing mode. In most studies, the direction of force could be placed into one of three categories: torque, axial load, or cantilever bending (varus or valgus). In addition to variations in the direction of force applied, a wide range of force magnitude and cycles were observed. For example, 33 studies used cyclic loading to test their constructs; however, the number of loading cycles used in each study ranged from 5 to 1,000,000 cycles. Furthermore, in many of these studies the magnitude of the force applied was not reported, or there was a wide variety in combinations of forces.

Similar heterogeneity was also observed in the reporting of cadaveric specimens used. Authors commonly did not report the age of the specimens or the pre-testing analysis conducted to ensure the validity of results. Only 57 % of studies reported the age of the specimens and 67 % reported their pre-testing analysis. Specifically, fewer than half of the studies reported the bone mineral density of their specimens, which is essential for ensuring testing specimens are comparable and the results can be interpreted within the context of other published studies.

The final gap identified in our scoping review was the lack of biomechanical testing of arthroplasty implants in proximal humerus fracture models. Although there are likely many studies that test the mechanical properties of shoulder arthroplasty implants in an intact humerus, only five studies were identified that performed testing within a PHF model. This lack of relevant testing is important to recognize because the implantation of a humeral arthroplasty stem in the setting of a proximal humerus fracture is technically challenging and inherently unstable due the displacement of the tuberosity fragments. Furthermore, given the exponential increase in reverse total shoulder arthroplasty for PHF patients, relevant biomechanical testing would provide invaluable information to help guide treatment decisions [2].

## Conclusion

The primary strength of this scoping review is the ability to identify key development areas to improve the quality and relevance of biomechanical modeling for proximal humerus fracture treatments. Our results suggest a strong need for implant testing in three- and four-part fracture models, testing of shoulder arthroplasty prostheses in a PHF model, and standardization of testing parameters to ensure results can be compared between studies. We anticipate this review will serve as springboard for designing studies aiming to address these key gaps in the future application of biomechanical testing for proximal humerus fracture treatments.

## Additional files

Additional file 1:
**Studies included in the analysis.** (DOCX 28 kb) Additional file 2:
**Implants tested in each included study.** (XLSX 46 kb)

## Abbreviations

CDSR: Cochrane database of systematic reviews
CINAHL: Cumulative Index of Nursing and Allied Health Literature
EMBASE: Excerpta medica database
IQR: Interquartile range
MeSH: Medical subject heading
PHF: Proximal humerus fracture
RTSA: Reverse total shoulder arthroplasty
SAE: Society of Automotive Engineers
TRID: Transport research international documentation
