# Supplementary material for: A scoping review of biomechanical testing for proximal humerus fracture implants
Source: BMC Musculoskelet Disord. 2015 Jul 30;16:175. doi: 10.1186/s12891-015-0627-x (PMC4520267; doi:10.1186/s12891-015-0627-x)
Supplement: Additional file 1: — Studies included in the analysis. (DOCX 28 kb) [file 12891_2015_627_MOESM1_ESM.docx]

**Appendix 1: Studies included in the analysis**

1. Bae JH, Oh JK, Chon CS, Oh CW, Hwang JH, Yoon YC. The biomechanical performance of locking plate fixation with intramedullary fibular strut graft augmentation in the treatment of unstable fractures of the proximal humerus. J Bone Joint Surg Br. 2011 Jul;93(7):937-41.
2. Brianza S, Plecko M, Gueorguiev B, Windolf M, Schwieger K. Biomechanical evaluation of a new fixation technique for internal fixation of three-part proximal humerus fractures in a novel cadaveric model. Clin Biomech (Bristol, Avon). 2010 Nov;25(9):886-92.
3. Brianza S, Röderer G, Schiuma D, Schwyn R, Scola A, Gebhard F, Tami AE. Where do locking screws purchase in the humeral head? Injury. 2012 Jun;43(6):850-5.
4. Brunner A, Resch H, Babst R, Kathrein S, Fierlbeck J, Niederberger A, Schmölz W. The Humerusblock NG: a new concept for stabilization of proximal humeral fractures and its biomechanical evaluation. Arch Orthop Trauma Surg. 2012 Jul;132(7):985-92.
5. Carrera EF, Nicolao FA, Netto NA, Carvalho RL, Dos Reis FB, Giordani EJ. A mechanical comparison between conventional and modified angular plates for proximal humeral fractures. J Shoulder Elbow Surg. 2008 Jul-Aug;17(4):631-6.
6. Castoldi F, Bonasia DE, Blonna D, Rossi R, Dettoni F, Assom M, Sankey A, Halewood C, Amis AA. The stability of percutaneous fixation of proximal humeral fractures. J Bone Joint Surg Am. 2010 Dec;92 Suppl 2:90-7.
7. Chow RM, Begum F, Beaupre LA, Carey JP, Adeeb S, Bouliane MJ. Proximal humeral fracture fixation: locking plate construct ± intramedullary fibular allograft. J Shoulder Elbow Surg. 2012 Jul;21(7):894-901.
8. Chudik SC, Weinhold P, Dahners LE. Fixed-angle plate fixation in simulated fractures of the proximal humerus: a biomechanical study of a new device. J Shoulder Elbow Surg. 2003 Nov-Dec;12(6):578-88.
9. De Wilde LF, Berghs BM, Beutler T, Ferguson SJ, Verdonk RC. A new prosthetic design for proximal humeral fractures: reconstructing the glenohumeral unit. J Shoulder Elbow Surg. 2004 Jul-Aug;13(4):373-80.
10. Dietz SO, Hartmann F, Schwarz T, Nowak TE, Enders A, Kuhn S, Hofmann A, Rommens PM. Retrograde nailing versus locking plate osteosynthesis of proximal humeral fractures: a biomechanical study. J Shoulder Elbow Surg. 2012 May;21(5):618-24.
11. Duda GN, Epari DR, Babst R, Lambert SM, Matthys R, Südkamp NP. Mechanical evaluation of a new minimally invasive device for stabilization of proximal humeral fractures in elderly patients: a cadaver study. Acta Orthop. 2007 Jun;78(3):430-5.
12. Durigan A Jr, Barbieri CH, Mazzer N, Shimano AC. Two-part surgical neck fractures of the humerus: mechanical analysis of the fixation with four Shanz-type threaded pins in four different assemblies. J Shoulder Elbow Surg. 2005 Jan-Feb;14(1):96-102.
13. Edwards SL, Wilson NA, Zhang LQ, Flores S, Merk BR. Two-part surgical neck fractures of the proximal part of the humerus. A biomechanical evaluation of two fixation techniques. J Bone Joint Surg Am. 2006 Oct;88(10):2258-64.
14. Eichhorn S, Grandl M, Trapp OM, Schreiber U. The poliax-nail; evaluation of a novel poly-axial and angle-stable intramedullary nailing concept. Injury. 2011 Sept;42(Supp 3):S21-22.
15. Erhardt JB, Stoffel K, Kampshoff J, Badur N, Yates P, Kuster MS. The position and number of screws influence screw perforation of the humeral head in modern locking plates: a cadaver study. J Orthop Trauma. 2012 Oct;26(10):e188-92.
16. Esen E, Doğramaci Y, Kömürcü M, Kanatli U, Bölükbaşi S, Atahan AO. Biomechanical comparison of fixation of two-part osteoporotic neck fracture of the proximal humerus using uni-planar and multi-planar Kirschner wire. Eklem Hastalik Cerrahisi. 2009;20(2):114-8.
17. Fankhauser F, Schippinger G, Weber K, Heinz S, Quehenberger F, Boldin C, Bratschitsch G, Szyszkowitz R, Georg L, Friedrich A. Cadaveric-biomechanical evaluation of bone-implant construct of proximal humerus fractures (Neer type 3). J Trauma. 2003 Aug;55(2):345-9.
18. Foruria AM, Carrascal MT, Revilla C, Munuera L, Sanchez-Sotelo J. Proximal humerus fracture rotational stability after fixation using a locking plate or a fixed-angle locked nail: the role of implant stiffness. Clin Biomech (Bristol, Avon). 2010 May;25(4):307-11.
19. Frankle MA, Greenwald DP, Markee BA, Ondrovic LE, Lee WE 3rd. Biomechanical effects of malposition of tuberosity fragments on the humeral prosthetic reconstruction for four-part proximal humerus fractures. J Shoulder Elbow Surg. 2001 Jul-Aug;10(4):321-6.
20. Frankle MA, Ondrovic LE, Markee BA, Harris ML, Lee WE 3rd. Stability of tuberosity reattachment in proximal humeral hemiarthroplasty. J Shoulder Elbow Surg. 2002 Sep-Oct;11(5):413-20.
21. Friedl W, Rinner M. L-F1. 1 Experimental examination of factors influencing the deformation cut out risk in locked nail osteosynthesis of proximal humerus fractures. *Injury*. 2012 Sep;*43*(Supp 1):S11.
22. Füchtmeier B, May R, Fierlbeck J, Hammer J, Nerlich M. A comparative biomechanical analysis of implants for the stabilization of proximal humerus fractures. Technol Health Care. 2006;14(4-5):261-70.
23. Gillespie RJ, Ramachandran V, Lea ES, Vallier HA. Biomechanical evaluation of 3-part proximal humerus fractures: a cadaveric study. Orthopedics. 2009 Nov;32(11):816. doi: 10.3928/01477447-20090922-06.
24. Gradl G, Knobe M, Stoffel M, Prescher A, Dirrichs T, Pape HC. Biomechanical evaluation of locking plate fixation of proximal humeral fractures augmented with calcium phosphate cement. J Orthop Trauma. 2013 Jul;27(7):399-404. doi: 10.1097/BOT.0b013e318278c595.
25. Gradl G, Stedtfeld HW, Morlock M, Sellenschloh K, Püschel K, Mittlmeier T, Gradl G. Locking plate fixation of humeral head fractures with a telescoping screw. A comparative biomechanical study versus a standard plate. Injury. 2012 Jun;43(6):734-8.
26. Hessmann MH, Hansen WS, Krummenauer F, Pol TF, Rommens P. Locked plate fixation and intramedullary nailing for proximal humerus fractures: a biomechanical evaluation. J Trauma. 2005 Jun;58(6):1194-201.
27. Horn J, Gueorguiev B, Brianza S, Steen H, Schwieger K. Biomechanical evaluation of two-part surgical neck fractures of the humerus fixed by an angular stable locked intramedullary nail. J Orthop Trauma. 2011 Jul;25(7):406-13.
28. Huffman GR, Itamura JM, McGarry MH, Duong L, Gililland J, Tibone JE, Lee TQ. Neer Award 2006: Biomechanical assessment of inferior tuberosity placement during hemiarthroplasty for four-part proximal humeral fractures. J Shoulder Elbow Surg. 2008 Mar-Apr;17(2):189-96.
29. Instrum K, Fennell C, Shrive N, Damson E, Sonnabend D, Hollinshead R. Semitubular blade plate fixation in proximal humeral fractures: a biomechanical study in a cadaveric model. J Shoulder Elbow Surg. 1998 Sep-Oct;7(5):462-6.
30. Jiang C, Zhu Y, Wang M, Rong G. Biomechanical comparison of different pin configurations during percutaneous pinning for the treatment of proximal humeral fractures. J Shoulder Elbow Surg. 2007 Mar-Apr;16(2):235-9.
31. Kedgley AE, DeLude JA, Drosdowech DS, Johnson JA, Bicknell RT. Humeral head translation during glenohumeral abduction following computer-assisted shoulder hemiarthroplasty. J Bone Joint Surg Br. 2008 Sep;90(9):1256-9.
32. Kitson J, Booth G, Day R. A biomechanical comparison of locking plate and locking nail implants used for fractures of the proximal humerus. J Shoulder Elbow Surg. 2007 May-Jun;16(3):362-6.
33. Koval KJ, Blair B, Takei R, Kummer FJ, Zuckerman JD. Surgical neck fractures of the proximal humerus: a laboratory evaluation of ten fixation techniques. J Trauma. 1996 May;40(5):778-83.
34. Kralinger F, Gschwentner M, Wambacher M, Smekal V, Haid C. Proximal humeral fractures: what is semi-rigid? Biomechanical properties of semi-rigid implants, a biomechanical cadaver based evaluation. Arch Orthop Trauma Surg. 2008 Feb;128(2):205-10.
35. Kralinger F, Unger S, Wambacher M, Smekal V, Schmoelz W. The medial periosteal hinge, a key structure in fractures of the proximal humerus: a biomechanical cadaver study of its mechanical properties. J Bone Joint Surg Br. 2009 Jul;91(7):973-6.
36. Kwon BK, Goertzen DJ, O'Brien PJ, Broekhuyse HM, Oxland TR. Biomechanical evaluation of proximal humeral fracture fixation supplemented with calcium phosphate cement. J Bone Joint Surg Am. 2002 Jun;84-A(6):951-61.
37. Lescheid J, Zdero R, Shah S, Kuzyk PR, Schemitsch EH. The biomechanics of locked plating for repairing proximal humerus fractures with or without medial cortical support. J Trauma. 2010 Nov;69(5):1235-42.
38. Lever JP, Aksenov SA, Zdero R, Ahn H, McKee MD, Schemitsch EH. Biomechanical analysis of plate osteosynthesis systems for proximal humerus fractures. J Orthop Trauma. 2008 Jan;22(1):23-9.
39. Liew AS, Johnson JA, Patterson SD, King GJ, Chess DG. Effect of screw placement on fixation in the humeral head. J Shoulder Elbow Surg. 2000 Sep-Oct;9(5):423-6.
40. Lill H, Hepp P, Korner J, Kassi JP, Verheyden AP, Josten C, Duda GN. Proximal humeral fractures: how stiff should an implant be? A comparative mechanical study with new implants in human specimens. Arch Orthop Trauma Surg. 2003 Apr;123(2-3):74-81.
41. Maldonado ZM, Seebeck J, Heller MO, Brandt D, Hepp P, Lill H, Duda GN. Straining of the intact and fractured proximal humerus under physiological-like loading. J Biomech. 2003 Dec;36(12):1865-73.
42. Mathison C, Chaudhary R, Beaupre L, Reynolds M, Adeeb S, Bouliane M. Biomechanical analysis of proximal humeral fixation using locking plate fixation with an intramedullary fibular allograft. Clin Biomech (Bristol, Avon). 2010 Aug;25(7):642-6.
43. Naidu SH, Bixler B, Capo JT, Moulton MJ, Radin A. Percutaneous pinning of proximal humerus fractures: a biomechanical study. Orthopedics. 1997 Nov;20(11):1073-6.
44. Onder U, Blauth M, Kralinger F, Schmoelz W. Shoulder joint abduction motion test bench: a new shoulder test bench for in vitro experiments with active muscle force simulation. Biomed Tech (Berl). 2012 May 30;57(3):163-8.
45. Osterhoff G, Baumgartner D, Favre P, Wanner GA, Gerber H, Simmen HP, Werner CM. Medial support by fibula bone graft in angular stable plate fixation of proximal humeral fractures: an in vitro study with synthetic bone. J Shoulder Elbow Surg. 2011 Jul;20(5):740-6.
46. Rajesh MB. The effect of bone quality on the intramedullary fixation of proximal humeral fractures. Bone. 2003 May;32(5):S165
47. Röderer G, Gebhard F, Krischak G, Wilke HJ, Claes L. Biomechanical in vitro assessment of fixed angle plating using a new concept of locking for the treatment of osteoporotic proximal humerus fractures. Int Orthop. 2011 Apr;35(4):535-41.
48. Rothstock S, Plecko M, Kloub M, Schiuma D, Windolf M, Gueorguiev B. Biomechanical evaluation of two intramedullary nailing techniques with different locking options in a three-part fracture proximal humerus model. Clin Biomech (Bristol, Avon). 2012 Aug;27(7):686-91.
49. Ruch DS, Glisson RR, Marr AW, Russell GB, Nunley JA. Fixation of three-part proximal humeral fractures: a biomechanical evaluation. J Orthop Trauma. 2000 Jan;14(1):36-40.
50. Saitoh S, Nakatsuchi Y. Osteoporosis of the proximal humerus: Comparison of bone-mineral density and mechanical strength with the proximal femur. J Shoulder Elbow Surg. 1993 Mar;2(2):78-84.
51. Saitoh S, Nakatsuchi Y, Latta L, Milne E. Distribution of bone mineral density and bone strength of the proximal humerus. J Shoulder Elbow Surg. 1994 Jul;3(4):234-42.
52. Sanders BS, Bullington AB, McGillivary GR, Hutton WC. Biomechanical evaluation of locked plating in proximal humeral fractures. J Shoulder Elbow Surg. 2007 Mar-Apr;16(2):229-34.
53. Schumer RA, Muckley KL, Markert RJ, Prayson MJ, Heflin J, Konstantakos EK, Goswami T. Biomechanical comparison of a proximal humeral locking plate using two methods of head fixation. J Shoulder Elbow Surg. 2010 Jun;19(4):495-501.
54. Sehr JR, Szabo RM. Semitubular blade plate for fixation in the proximal humerus. J Orthop Trauma. 1988;2(4):327-32.
55. Seide K, Triebe J, Faschingbauer M, Schulz AP, Püschel K, Mehrtens G, Jürgens Ch. Locked vs. unlocked plate osteosynthesis of the proximal humerus - a biomechanical study. Clin Biomech (Bristol, Avon). 2007 Feb;22(2):176-82.
56. Siffri PC, Peindl RD, Coley ER, Norton J, Connor PM, Kellam JF. Biomechanical analysis of blade plate versus locking plate fixation for a proximal humerus fracture: comparison using cadaveric and synthetic humeri. J Orthop Trauma. 2006 Sep;20(8):547-54.
57. Tingart MJ, Lehtinen J, Zurakowski D, Warner JJ, Apreleva M. Proximal humeral fractures: regional differences in bone mineral density of the humeral head affect the fixation strength of cancellous screws. J Shoulder Elbow Surg. 2006 Sep-Oct;15(5):620-4.
58. Unger S, Erhart S, Kralinger F, Blauth M, Schmoelz W. The effect of in situ augmentation on implant anchorage in proximal humeral head fractures. Injury. 2012 Oct;43(10):1759-63.
59. Vogel G, Chechick A, Pritch Perry M, Brosh T. Fixation of humeral surgical neck fracture using contoured pins versus straight pins: a mechanical study. Int Orthop. 2007 Dec;31(6):811-5.
60. Voigt C, Hurschler C, Rechi L, Vosshenrich R, Lill H. Additive fiber-cerclages in proximal humeral fractures stabilized by locking plates: no effect on fracture stabilization and rotator cuff function in human shoulder specimens. Acta Orthop. 2009 Aug;80(4):465-71.
61. Voigt C, Kreienborg S, Megatli O, Schulz AP, Lill H, Hurschler C. How does a varus deformity of the humeral head affect elevation forces and shoulder function? A biomechanical study with human shoulder specimens. J Orthop Trauma. 2011 Jul;25(7):399-405.
62. Wallace MJ, Bledsoe G, Moed BR, Israel HA, Kaar SG. Relationship of cortical thickness of the proximal humerus and pullout strength of a locked plate and screw construct. J Orthop Trauma. 2012 Apr;26(4):222-5.
63. Weinstein DM, Bratton DR, Ciccone WJ 2nd, Elias JJ. Locking plates improve torsional resistance in the stabilization of three-part proximal humeral fractures. J Shoulder Elbow Surg. 2006 Mar-Apr;15(2):239-43.
64. Wheeler DL, Colville MR. Biomechanical comparison of intramedullary and percutaneous pin fixation for proximal humeral fracture fixation. J Orthop Trauma. 1997 Jul;11(5):363-7.
65. Williams GR Jr, Copley LA, Iannotti JP, Lisser SP. The influence of intramedullary fixation on figure-of-eight wiring for surgical neck fractures of the proximal humerus: a biomechanical comparison. J Shoulder Elbow Surg. 1997 Sep-Oct;6(5):423-8.
66. Wychowański M, Obrebski M, Rapała K, Wit A, Gajewski J, Marczak K. Strength of proximal humeral fraction fixation employing implants of various types--a study of porcine bones. Acta Bioeng Biomech. 2008;10(3):29-35.
67. Zettl R, Müller T, Topp T, Lewan U, Krüger A, Kühne C, Ruchholtz S. Monoaxial versus polyaxial locking systems: a biomechanical analysis of different locking systems for the fixation of proximal humeral fractures. Int Orthop. 2011 Aug;35(8):1245-50.
